# Comparison of clinical outcomes between intravascular optical coherence tomography-guided and angiography-guided stent implantation: A meta-analysis of randomized control trials and systematic review: Retraction

**DOI:** 10.1097/MD.0000000000017379

**Published:** 2019-09-20

The article, “Comparison of clinical outcomes between intravascular optical coherence tomography-guided and angiography-guided stent implantation: A meta-analysis of randomized control trials and systematic review”,^[1]^ which appeared in Volume 98, Issue 6 of *Medicine*, is being retracted by the journal.

The authors included 11 studies in the meta-analysis, however, two of them have the same study population and multiple patients were duplicated in the research. In addition, the first study criteria needed to be altered to “patients were underwent PCI using a metallic drug-eluting stent (DES), bioresorbable vascular scaffolds or metallic BMS” which would alter the sample size and affect the conclusion of the study.
